# Comments on introducing the immune system

**DOI:** 10.1186/1753-4631-3-3

**Published:** 2009-06-11

**Authors:** E Ahmed

**Affiliations:** 1 Mathematics department, Faculty of Science, Mansoura 35516, Egypt

## Abstract

It is argued that by studying some design principles of the immune system, e.g. nonlinearity and being a complex adaptive system, one can easily find some explanations of basic properties of the system e.g. memory and tolerance.

## Introduction

Most of the immunology books treat this subject as mainly an experimental science. Within few pages the reader is faced with many cells, secreting many molecules each doing many functions. Also each function is done by many immune effectors. Little attention is made to the design principles of the immune system (IS). Even some prominent immunologists have expressed the same concern [1]. Here we attempt to discuss some design principles of IS.

### Immune systenm and Complex adaptive systems (CAS) [2-4]

CAS consists of adaptive (capable of learning) entities. Some properties of such systems cannot be understood just by studying the constituents. They are called emergent properties.

Examples of CAS include the brain where the emergent property is cognition which is a property of the brain not of individual cells. We propose that IS is a CAS with tolerance as an emergent property since tolerance is a property of IS as a whole not individual B or T or macrophage etc.... cells. In fact almost every biological, social and financial system is a CAS.

Emergent properties implies that reductions approach is not suitable for CAS. Hence these systems have to be studied as a whole in addition to the reductionest approach. It is crucial to see that both approaches complement each other and are not contradictory [5].

CAS have some generic properties e.g. they are open (connected to other systems). Interactions in CAS are nonlinear hence long range predictions in CAS are highly unlikely. Typically they have a network (e.g. evolving network) structure. Optimization in such systems is multiobjective. Control of most CAS is difficult, it should be targeted and integrated i.e. using diverse approaches not just one. Most CAS show delay and memory behavior. Spatial effects should not be neglected in many CAS. Most, but not all, CAS are decentralized.

CAS should be studied as a whole in addition to studying its constituents. Hence in vivo experiments are crucial to understand them.

IS consists of adaptive interacting cells.

IS has a network structure. Moreover it is a threshold network c.f. high and low zone tolerance of IS.

Tolerance is an example of an emergent property since it cannot be attributed to the immune effectors only but the infection tissue and the Antigen (Ag) quantity are relevant factors.

Hence IS is a CAS.

### Immune system engineering

Here IS is considered from the design point of view i.e. one will determine the objectives of IS then try to find a way to achieve them. We do not claim that these are the only way to achieve these objectives. However, judging by the millions of years in which IS is capable of protecting animals and humans and adding the huge variety of the Ag from bacteria and viruses to chemicals to harmful waves etc ..its design is nothing short of a miracle.

Basic objectives of IS are to protect the body against harmful antigens (Ag). And to use the bounded resources of the body efficiently.

To achieve the first objective IS has to be able to recognize a huge number of Ag. This cannot be done by a single organ hence IS has to be decentralized.

This ability to recognize the huge number of Ag implies the danger of attacking useful organs in the body (autoimmunity (AIm)) hence mechanisms have to exist to control this danger. Such mechanisms include the central clonal deletion in the thymus and bone marrow, the high and low zone tolerance of the immune network and the two signals required for activating immune B and T cells.

To achieve the second goal, more efficient immune response should exist to recurrent Ag hence IS should contain a part which memorizes recurring Ag. Thus IS contains two parts one nonspecific and quick to react while the other is specific and, a bit, slow to react but it is more efficient against recurrent Ag.

IS has to be controlled hence both positive and negative mechanisms have to exist with negative ones dominating positive ones to prevent excessive immune response that may harm the individual (c.f. the case of anaphylaxes where immune response may cause death).

## Some conclusions

Targeted control for IS implies that in transplantations it may be better to block signal II instead of signal I. Several immunologists have reached this conclusion some years ago.

IS dynamics is called extremal dynamics (clones which are activated live longer while other clones die of neglect). It is known [6,7] that such dynamics lead to an explanation for the long memory of IS without Ag persistence since this dynamics lead to the following relation: The probability of removal of a clone which has lived for time t is proportional to 1/(t+c) where c is a positive constant.

Integrated approaches for treatments imply that immunotherapy of cancer needs to be augmented by other treatment method. Also multi-drugs with independent modes of action should be used.

IS is a multi-functional multi-pathway system hence total failure of IS is rare.

In vivo experiments are significantly more reliable than in vitro ones to understand IS.

IS indicates some interesting topics mathematically e.g. decentralized systems, fractional order mathematics and extremal optimization etc.. Hence studying IS is quite interesting medically, biologically, physically, chemically, for computer scientists and even mathematically. The problem of protecting the data which faces computer scientists is almost identical to the one which IS has succeeded to solve. One expects that different approaches to the study of IS will be useful.

There is an extraordinary similarity between IS and insect swarms. Some of the principles of insect collective behavior includes [8]: Non-selfish key individuals, positive and negative feed back, diversity and threshold responses. All

these characteristics exist in IS e.g. Th cells, high and low zone tolerance and the fact that most immune effectors have more than one function and almost all immune functions are done by more than one effector hence diversity is intrinsic in IS.

### Some open questions

IS initiation is an important problem. Presently two theorems are proposed self-nonself and danger theory [9]. Danger theory solves some problems in self-nonself theory e.g. lactation, pregnancy but is the correct theory one of them or a hybrid of them?

If danger is the only mechanism for IS initiation then what is the difference between autoimmunity and hypersensitivity? After all both are pathogenic immune responses to, normally, tolerated substances and cells.

The rejection of the second pregnancy due to Rh differences between fetus and mother, one thinks, poses a serious problem to self-nonself theory. But why the second pregnancy and not the first one is rejected. An answer is proposed that the danger signals result from the first child "birth". But one thinks that in this case the way of the birth (whether normal or cesarean) is expected to affect the duration of the second pregnancy before it fails (normal first birth gives a longer duration for the second pregnancy duration). Does agree or disagree with observation?

In anaphylaxes two mechanisms seem to be broken, the first is tolerance and the second is the control mechanism of the immune response. The immune response in the case of anaphylaxes may cause death!! The simultaneous breakdown of two mechanisms seems quite puzzling.

It is important to understand that what is proposed here does not replace the traditional approach to IS. It is proposed that few minutes spent on the design principles of IS may be helpful in trying to understand the huge amount of details that exists in IS.

## Competing interests

The author declares that they have no competing interests.
